# U-shaped association between serum uric acid at admission and post-stroke epilepsy in patients with ischemic stroke: a cohort study

**DOI:** 10.3389/fneur.2026.1759537

**Published:** 2026-02-19

**Authors:** Dayuan Liu, Hao Peng, Yihao Zhai, Muyao Wang, Hongli Jiang, Baoshou Su, Yunxiang Zhong, Guolong Deng, Ning Li, Jigao Feng, Caicai Zhang

**Affiliations:** 1 Department of Neurosurgery, The Second Affiliated Hospital of Hainan Medical University, Haikou, Hainan, China; 2 Key Laboratory of Tropical Translational Medicine of Ministry of Education & Key Laboratory of Brain Science Research Transformation in Tropical Environment of Hainan Province, School of Basic Medicine and Life Sciences, Hainan Medical University, Haikou, Hainan, China; 3 Department of Neurosurgery, The Second People’s Hospital of Hainan Province, Wuzhishan, Hainan, China; 4 Department of Neurosurgery, Hainan Affiliated Hospital of Hainan Medical University, Haikou, Hainan, China

**Keywords:** inflammation, ischemic stroke, oxidative stress, post-stroke epilepsy, serum uric acid, U-shaped association

## Abstract

**Background:**

The relationship between serum uric acid (SUA) and post-stroke epilepsy (PSE) remains uncertain. We investigated the association between serum uric acid at admission and PSE after acute ischemic stroke.

**Methods:**

A retrospective cohort study was conducted on 21,459 ischemic stroke patients. Serum uric acid at admission was measured as part of routine laboratory testing and analyzed in quartiles (Q1-Q4). Logistic regression models with restricted cubic splines (RCS) were used to investigate the potential nonlinearity of the association between serum uric acid (SUA) levels and the risk of post-stroke epilepsy (PSE), with adjustments for demographic, clinical, and laboratory variables. Propensity score matching (PSM) was additionally employed to address potential confounding.

**Results:**

A U-shaped association was observed. Compared with the mid-range (Q2–Q3: ~305.3–373.9 μmol/L), both low (Q1) and high (Q4) SUA were associated with higher PSE risk (adjusted OR 2.32, 95% CI 1.79–2.99; and 1.60, 95% CI 1.26–2.02). RCS identified an inflection point around 335 μmol/L: below this level, higher SUA related to lower PSE risk, whereas above it, higher SUA related to higher risk. Findings were robust in propensity score–based and sensitivity analyses.

**Conclusion:**

SUA shows a U-shaped association with PSE after ischemic stroke. SUA levels within an intermediate range were associated with a lower risk of PSE. This observation is hypothesis-generating and requires confirmation in prospective studies to evaluate potential causal relationships.

## Introduction

Stroke ranks as the second-leading cause of mortality worldwide, frequently accompanied by severe complications during both its acute and recovery stages (1). Post-stroke epilepsy (PSE) affects approximately 5–9% of patients after stroke (2). It is widely recognized that PSE contributes to extended hospitalization durations and escalates mortality rates in cerebrovascular disease patients. Ischemic stroke has been identified as the predominant cause of PSE, accounting for 70–85% of cases associated with stroke-related epilepsy (3). The reported frequency of post-stroke ictal and interictal epileptiform activity after stroke is 7 and 8%, respectively (4). Addressing the profound influence of PSE on patient prognosis necessitates a thorough investigation of its risk factors, which is vital for enabling precise risk prediction and for generating hypotheses that may inform future preventive and therapeutic research (5).

Recent studies have highlighted serum uric acid (SUA) as a biomarker of growing importance in stroke and PSE research (5, 6). SUA, a metabolic byproduct of purine degradation, exhibits contrasting roles within the nervous system. While moderate levels exert antioxidant properties that counter neuronal damage caused by oxidative stress, elevated SUA concentrations may act as pro-oxidants, intensifying inflammatory responses and amplifying neural excitability (7). Previous research has revealed a “U-shaped” dose–response correlation between SUA levels and ischemic stroke risk (8), emphasizing that both exceedingly high and low SUA levels markedly elevate stroke risk (9, 10). This dualistic behavior suggests SUA’s complex involvement in stroke pathophysiology, wherein it provides protective effects at optimal levels but contributes to adverse outcomes at extremes (11). Nonetheless, the association between SUA levels and epilepsy risk remains contentious. Certain studies propose a “L-shaped” relationship, arguing that lower SUA levels heighten epilepsy risk, whereas higher levels seemingly plateau without further impact (12). Supporters of this view underscore the significance of SUA’s antioxidant activity, asserting that insufficient levels heighten vulnerability to oxidative damage, thereby fostering epileptogenesis (13). Contrarily, alternative findings link elevated SUA to an increased likelihood of late-onset seizures post-ischemic stroke, implicating oxidative stress and inflammatory cascades as underlying mechanisms (14, 15). These divergent results imply that SUA’s role in PSE may vary depending on disease stage and individual patient conditions.

In the specific context of ischemic stroke, the interaction between SUA and PSE emerges as particularly intricate and remains insufficiently understood. For instance, low SUA levels may undermine antioxidant defenses, whereas excessive levels could aggravate inflammation and neuronal injury, collectively elevating epilepsy risks (16). This dual role accentuates the need for elucidating SUA’s biological mechanisms in PSE. Accordingly, the present study endeavors to systematically explore the association between SUA levels and PSE in ischemic stroke patients. By analyzing these relationships comprehensively, this study aims to provide foundational evidence supporting the prediction and clinical prevention of PSE.

## Materials and methods

### Data source

The dataset for this analysis was sourced from the Dryad Digital Repository.^1^ Initially, this dataset was part of a study focusing on predictive models for post-stroke epilepsy in patients with acute ischemic stroke (17). Its availability for public use was granted by the original authors, enabling secondary analysis while adhering to ethical research standards, including compliance with STROBE guidelines and Dryad’s Terms of Service.

### Study population

This investigation included a total of 21,459 patients diagnosed with acute ischemic stroke who were admitted to Chongqing Emergency Center over a five-year period (June 2017–June 2022). Patients aged between 18 and 90 years were eligible for inclusion, provided they had no prior history of stroke, epilepsy, or related conditions, and were not deceased within 72 h or 3 months post-stroke. Individuals lost to follow-up were likewise excluded. Data collection encompassed imaging studies (CT/MRI, CTA/MRA/DSA) and laboratory assessments, including blood lipid profiles, liver and kidney function markers, coagulation indicators, and myocardial enzyme levels. Secondary analyses utilized de-identified data from the Dryad Digital Repository (17).

### Baseline serum uric acid

Serum uric acid at admission was measured as part of routine laboratory testing and analyzed as quartiles (Q1–Q4).

### Covariates

Key covariates for regression analyses were selected based on clinical relevance, statistical significance in preliminary testing, and alignment with study objectives. These included demographic data (age, sex, and NIHSS scores at admission), comorbidities (e.g., hypertension, diabetes, atrial fibrillation, coronary disease, uremia, deep vein thrombosis, fatty liver, cerebral herniation, hydrocephalus, hypoproteinemia, and hyperlipidemia), vascular involvement (stenosis or occlusion of major cerebral arteries verified via CTA/MRA/DSA), and laboratory parameters such as red blood cell count, platelet count, renal function, low-density lipoprotein cholesterol (LDL-C) levels, and fibrinogen concentration. This systematic approach ensured a balance between clinical utility and statistical robustness, minimizing risks of overfitting.

### Outcome

The primary outcome was PSE within 1 year after acute ischemic stroke, defined as the pre-labeled PSE variable provided in the Dryad dataset (see text footnote 1) derived from the published predictive-model study.

### Statistical analysis

Descriptive analyses were performed for all patient data. Continuous variables were expressed as mean ± standard deviation for normally distributed data or median with interquartile range for skewed data. Categorical variables were reported as counts and percentages. Comparisons between categorical variables were assessed using chi-square or Fisher’s exact tests, whereas continuous variables were evaluated through one-way ANOVA or Kruskal-Wallis tests, depending on their distributions. Logistic regression models were employed to examine the association between SUA levels and PSE risk, with odds ratios (ORs) and 95% confidence intervals (CIs) reported for unadjusted (Model 1), age- and sex-adjusted (Model 2), and fully adjusted analyses (Model 3). We further performed sex-stratified analyses and tested effect modification by including a SUA quartiles × sex interaction term. The interaction was evaluated using a likelihood ratio test comparing models with and without the interaction term. We assessed collinearity using the variance inflation factor (VIF), with values greater than 10 indicating problematic collinearity (18). Nonlinearity in continuous variables was modeled using restricted cubic splines with four knots placed at the 5th, 35th, 65th, and 95th percentiles (19). To address potential imbalances in covariates, propensity score matching (PSM) was performed using logistic regression with a logit link to estimate propensity scores (R package ‘MatchIt’) (20). One-to-one nearest-neighbor matching without replacement was applied, with the caliper width set to 0.2 of the standard deviation of the logit of the propensity score. Covariate balance after matching was assessed using standardized mean differences (SMDs), with values below 0.2 indicating adequate balance (21). Sensitivity analyses excluded patients with uremia, hydrocephalus, or cerebral herniation to ensure the robustness of findings. Statistical analyses were conducted using R software (version 4.4.1), with significance defined at two-sided *p*-value < 0.05.

## Results

### Baseline characteristics of participants

The baseline characteristics of the participants are summarized in Table 1. A total of 21,459 patients were included in the study, with an average age of 66.41 ± 12.35 years. Among them, 49.47% were female and 50.53% were male. Participants were divided into quartiles (Q1-Q4) based on serum uric acid (SUA) levels, with average SUA levels of 279.31 ± 24.05 μmol/L, 320.85 ± 8.57 μmol/L, 351.28 ± 10.86 μmol/L, and 425.28 ± 41.18 μmol/L, respectively. Participants aged ≥60 years accounted for 73.68% of the cohort, and NIH Stroke Scale (NIHSS) scores averaged 8.01 ± 2.94. Most participants (73.31%) had NIHSS scores <10. Key comorbidities included hypertension (68.74%), diabetes (34.12%), coronary disease (45.07%), and hyperlipidemia (20.83%). The prevalence of PSE was 4.36%, with significant differences across SUA quartiles (*p* < 0.0001). Laboratory indicators showed significant differences across quartiles, including platelet count, red blood cell count, LDL-C, creatinine, urea, and fibrinogen.

**Table 1:** Baseline characteristics of participants by quartiles of blood uric acid.^a^

Characteristics	Total	Q1 (<305.3)	Q2 (305.3–335.3)	Q3 (335.3–373.9)	Q4 (≥373.9)	*p*-value
	(*n* = 21,459)	(*n* = 5,372)	(*n* = 5,374)	(*n* = 5,349)	(*n* = 5,364)	
Blood uric acid, μmol/L	344.14 ± 58.84	279.31 ± 24.05	320.85 ± 8.57	351.28 ± 10.86	425.28 ± 41.18	<0.0001
Age (years)	66.41 ± 12.35	68.26 ± 12.14	67.08 ± 11.81	65.04 ± 11.75	65.25 ± 13.35	<0.0001
Age						<0.0001
<60	5,647 (26.32)	1,095 (20.38)	1,315 (24.47)	1,468 (27.44)	1769 (32.98)	
> = 60	15,812 (73.68)	4,277 (79.62)	4,059 (75.53)	3,881 (72.56)	3,595 (67.02)	
Gender						<0.0001
Female	10,616 (49.47)	4,182 (77.85)	3,261 (60.68)	1732 (32.38)	1,441 (26.86)	
Male	10,843 (50.53)	1,190 (22.15)	2,113 (39.32)	3,617 (67.62)	3,923 (73.14)	
NIHSS	8.01 ± 2.94	8.91 ± 3.27	8.12 ± 3.18	7.77 ± 2.49	7.24 ± 2.45	<0.0001
NIHSS						<0.0001
<10	15,731 (73.31)	4,181 (78.16)	3,286 (61.17)	3,715 (69.13)	4,549 (84.81)	
≥10	5,728 (26.69)	1,168 (21.84)	2086 (38.83)	1,659 (30.87)	815 (15.19)	
Uremia	190 (0.89)	11 (0.20)	19 (0.35)	23 (0.43)	137 (2.55)	<0.0001
Deep vein thrombosis	1,324 (6.17)	448 (8.34)	312 (5.81)	230 (4.30)	334 (6.23)	<0.0001
Fatty liver	4,245 (19.78)	560 (10.42)	1,044 (19.43)	1,027 (19.20)	1,614 (30.09)	<0.0001
Diabetes	7,322 (34.12)	1,087 (20.23)	1748 (32.53)	2047 (38.27)	2,440 (45.49)	<0.0001
Hypertension	14,751 (68.74)	3,274 (60.95)	3,472 (64.61)	3,605 (67.40)	4,400 (82.03)	<0.0001
Coronary disease	9,672 (45.07)	1993 (37.10)	2065 (38.43)	2,565 (47.95)	3,049 (56.84)	<0.0001
Atrial fibrillation	2043 (9.52)	460 (8.56)	421 (7.83)	496 (9.27)	666 (12.42)	<0.0001
Cerebral herniation	181 (0.84)	86 (1.60)	25 (0.47)	42 (0.79)	28 (0.52)	<0.0001
Hydrocephalus	281 (1.31)	78 (1.45)	85 (1.58)	62 (1.16)	56 (1.04)	0.05
Hyperlipidemia	4,469 (20.83)	974 (18.13)	1,115 (20.75)	1,000 (18.70)	1,380 (25.73)	<0.0001
Hypoproteinemia	2,421 (11.28)	1,027 (19.12)	418 (7.78)	493 (9.22)	483 (9.00)	<0.0001
Anterior cerebral artery	274 (1.28)	120 (2.23)	63 (1.17)	37 (0.69)	54 (1.01)	<0.0001
Middle cerebral artery	828 (3.86)	198 (3.69)	196 (3.65)	225 (4.21)	209 (3.90)	0.42
Posterior cerebral artery	62 (0.29)	17 (0.32)	21 (0.39)	13 (0.24)	11 (0.21)	0.29
Vertebral artery	652 (3.04)	183 (3.41)	158 (2.94)	135 (2.52)	176 (3.28)	0.04
Basilar artery	191 (0.89)	75 (1.40)	37 (0.69)	29 (0.54)	50 (0.93)	<0.0001
Platelet count, (×10^9^/L)	189.90 ± 27.02	188.02 ± 30.01	190.00 ± 24.67	188.81 ± 23.84	192.76 ± 28.81	<0.0001
Red blood cell count, (×10^12^/L)	4.31 ± 0.32	4.18 ± 0.27	4.25 ± 0.28	4.40 ± 0.30	4.41 ± 0.36	<0.0001
LDL-C, mg/dL	2.69 ± 0.36	2.65 ± 0.40	2.70 ± 0.31	2.70 ± 0.36	2.71 ± 0.36	<0.0001
Creatinine, μmol/L	84.89 ± 51.17	70.55 ± 14.23	76.99 ± 18.60	79.67 ± 26.10	112.35 ± 90.53	<0.0001
Urea, mmol/L	6.40 ± 1.43	6.05 ± 0.98	6.27 ± 1.11	6.31 ± 1.41	6.98 ± 1.88	<0.0001
Fibrinogen, g/L	3.60 ± 0.47	3.56 ± 0.54	3.55 ± 0.39	3.57 ± 0.42	3.72 ± 0.51	<0.0001
post-stroke epilepsy	936 (4.36)	314 (5.85)	220 (4.09)	156 (2.92)	246 (4.59)	<0.0001

a Continuous variables are presented as mean±SD or median (IQR); categorical variables are presented as *n* (%).

NIHSS, National Institutes of Health Stroke Scale.

### Association of SUA and PSE

Table 2 illustrates the association between serum uric acid (SUA) levels and post-stroke epilepsy (PSE) across three logistic regression models. In Model 3, all variance inflation factor (VIF) values were below 5, indicating that multicollinearity did not significantly affect the model’s stability (Supplementary Table S1). In all models, both the lowest SUA quartile (Q1) and the highest SUA quartile (Q4) were significantly associated with increased risks of PSE compared to the reference group (Q3). After considering potential confounders, a U-shaped relationship between SUA and PSE was observed through quartile analysis. Compared to the Q3 group, the odds ratios (ORs) for Q1, Q2 and Q4 were 2.32 (95% CI: 1.79–2.99, *p* < 0.0001), 1.5 (95% CI: 1.18–1.92, *p* = 0.001) and 1.60 (95% CI: 1.26–2.02, p < 0.0001), respectively. Considering the comparable risks observed in Q2 and Q3, the second and third quartiles were combined into one group (the 2–3 quartiles) for simplified analysis. As expected, compared with those in the 2–3 quartiles, participants in Q1 and Q4 demonstrated significantly higher risks of PSE, with adjusted ORs of 1.80 (95% CI: 1.47–2.22) and 1.35 (95% CI: 1.10–1.66) in the fully adjusted Model 3, respectively. To further explore sex differences, we conducted sex-stratified analyses of the association between SUA quartiles and PSE risk (Q3 as the reference). Among women, the ORs for Q1, Q2, and Q4 versus Q3 were 1.074 (95% CI 0.794–1.472; *p* = 0.651), 0.838 (95% CI 0.602–1.176; *p* = 0.299), and 1.341 (95% CI 0.934–1.931; *p* = 0.112), respectively. Among men, the corresponding ORs were 5.740 (95% CI 4.436–7.463; *p* < 0.0001), 2.315 (95% CI 1.772–3.035; *p* < 0.0001), and 1.747 (95% CI 1.364–2.250; *p* < 0.0001) (Supplementary Table S2). A likelihood ratio test indicated a significant interaction between SUA quartiles and sex (LR *χ*^2^ = 104.83; Δdf = 3; P for interaction < 2.2 × 10^−16^) (Supplementary Table S3).

**Table 2:** Associations between serum uric acid levels and epilepsy.

Serum uric acid, μmol/L	Model 1	*p*-value	Model 2	*p*-value	Model 3	*p*-value
	OR (95%CI)		OR (95%CI)		OR (95%CI)	
Quartiles						
Q1	2.07 (1.70, 2.51)	<0.0001	2.96 (2.41, 3.65)	<0.0001	2.32 (1.79, 2.99)	<0.0001
Q2	1.42 (1.15, 1.75)	<0.001	1.77 (1.43, 2.18)	<0.0001	1.50 (1.18, 1.92)	0.001
Q3	Ref		Ref		Ref	
Q4	1.60 (1.30, 1.96)	<0.0001	1.53 (1.25, 1.88)	<0.0001	1.60 (1.26, 2.02)	<0.0001
Categorical						
Q1	1.71 (1.47, 1.99)	<0.0001	2.15 (1.83, 2.53)	<0.0001	1.80 (1.47, 2.22)	<0.0001
Q2-Q3	Ref		Ref		Ref	
Q4	1.32 (1.12, 1.56)	<0.001	1.16 (0.98, 1.37)	<0.08	1.35 (1.10, 1.66)	0.005

Model 1: No covariates were adjusted.

Model 2: Age, gender.

Model 3: Age, gender, NIHSS, Uremia, Deep vein thrombosis, Fatty liver, Diabetes, Hypertension, Coronary disease.

Atrial fibrillation, Cerebral herniation, Hydrocephalus, Hyperlipidemia, Hypoproteinemia, Anterior cerebral artery.

Middle cerebral artery, Posterior cerebral artery, Vertebral artery, Basilar artery, Platelet count, Red blood cell count.

LDL-C, Creatinine, Urea, and Fibrinogen.

Q, quartiles; OR, odds ratio; 95% CI, 95% confidence interval.

### Restricted cubic spline (RCS) analysis

**Figure 1:**
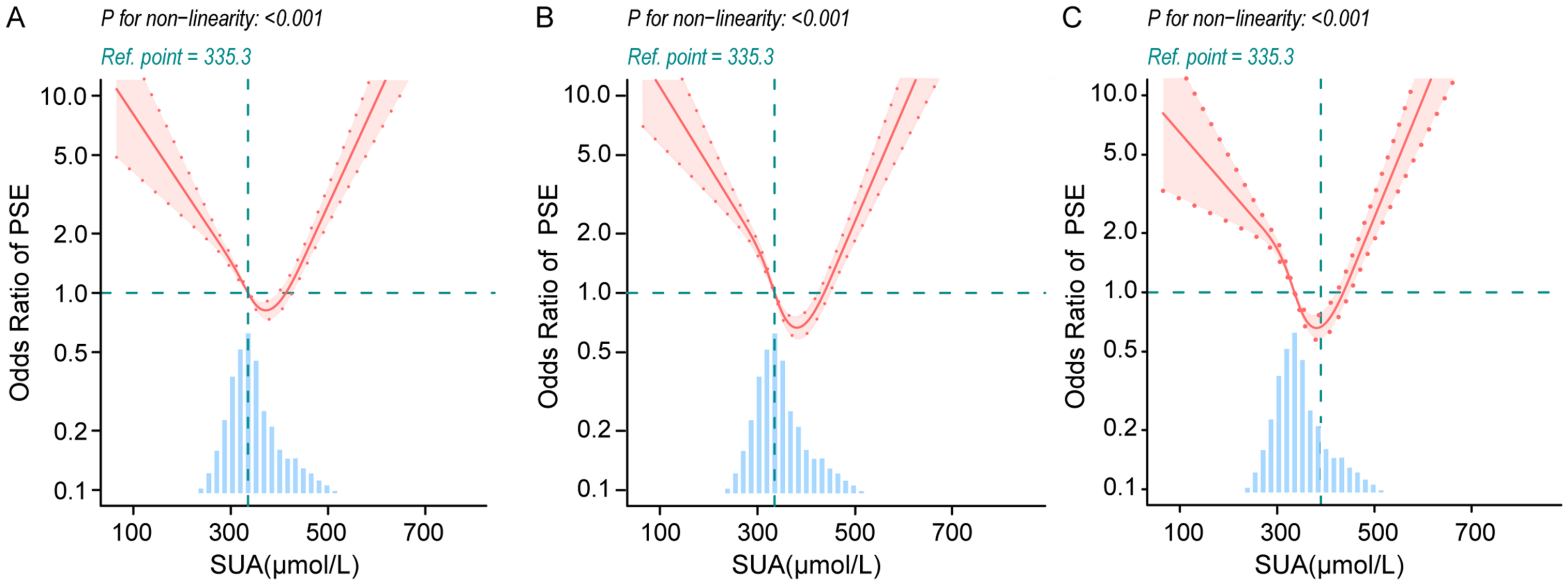
Nonlinear association between serum uric acid at admission and post-stroke epilepsy (PSE) in restricted cubic spline models. The solid red curve denotes the estimated odds ratio (OR) and the shaded area the 95% confidence interval; the horizontal dashed line indicates OR = 1, and the vertical dashed line marks the reference SUA level (335.3 μmol/L). The histogram below shows the distribution of SUA **(A)** Unadjusted model. **(B)** Model adjusted for age and sex. **(C)** Fully adjusted model including age, sex, NIHSS at admission; comorbidities (uremia, deep vein thrombosis, fatty liver, diabetes, hypertension, coronary disease, atrial fibrillation, cerebral herniation, hydrocephalus, hyperlipidemia, hypoproteinemia); major cerebral artery stenosis/occlusion (anterior, middle, posterior cerebral; vertebral; basilar); and laboratory measures (platelet count, red blood cell count, LDL-C, creatinine, urea, fibrinogen). Nonlinearity was assessed with a likelihood ratio test; *p* < 0.001 for all panels.

In our multivariate Logistic regression analysis, we identified a possible nonlinear association between the SUA levels and PSE risk. To further validate the association between the SUA levels and PSE risk, we employed RCS analysis. Our results demonstrated a U-shaped association between the SUA levels and PSE risk in Models 1, 2 and 3 (*p* value for nonlinearity <0.001) (Figure 1). In addition to this, we further fitted the association between the serum uric acid levels at admission and PSE risk using a two-piecewise multivariate logistic regression model and found two different slopes. We identified an inflection point at approximately 335.3 μmol/L (Figure 1). Below this threshold (SUA < 335.3 μmol/L), SUA levels were negatively associated with PSE risk, while above this threshold, they were positively associated. On the left side of the inflection point, the OR was 0.992 (95% CI: 0.989–0.996, *p* < 0.001). On the right side of the inflection point, the ORs was 1.009 (95% CI: 1.007–1.012, *p* < 0.0001) (Table 3). This indicates that the risk of PSE started to decrease by 0.8% per 1 μmol/L SUA change until a level of 335.3 μmol/L. Then the risk of PSE started to increase by 0.9% per 1 μmol/L SUA change (*p*-value for non-linearity <0.0001).

**Table 3:** Threshold effect of serum uric acid levels on secondary epilepsy risk in acute ischemic stroke patients.^a^

Epilepsy	OR (95%CI)	*p*-value
Fitting by the standard linear regression	1.002 (1.001,1.004)	0.003
Fitting model by two-piecewise linear regression		
Inflection point	335.3	
SUA < IP	0.992 (0.989,0.996)	<0.001
SUA ≥ IP	1.009 (1.007, 1.012)	<0.0001
p for Log-likelihood ratio		<0.0001

a Adjusted for all covariates in Table 1.

SUA, serum uric acid; OR, odds ratio; 95% CI, 95% confidence interval; IP, Inflection point.

### Analysis of the association between SUA and PSE after PSM

To simplify analysis, the Q2 and Q3 groups (quartiles 2–3, 305.3 to 373.9 μmol/L) were combined due to their comparable risks of PSE. Propensity score matching (PSM) significantly improved the balance of covariates between comparison groups, as demonstrated in Supplementary Table S1. Most variables achieved adequate balance with standardized mean differences (SMDs) below 0.2 after matching, except for Creatinine and Urea, which slightly exceeded the commonly accepted threshold (Supplementary Figure S2). Despite this residual imbalance, propensity score-matched logistic regression models (both unadjusted and adjusted) consistently demonstrated a U-shaped association between serum uric acid (SUA) levels and PSE risk. Compared to the Q2–Q3 group, participants in Q1 showed an adjusted odds ratio (OR) of 1.94 (95% CI: 1.56–2.42), while those in Q4 exhibited an adjusted OR of 1.33 (95% CI: 1.02–1.74) (Table 4). Furthermore, RCS analyses supported the U-shaped association across all three models (Model 1, Model 2, and Model 3) in the post-PSM dataset, with *p* values for nonlinearity consistently <0.001 (Supplementary Figure S2).

**Table 4:** Association between serum uric acid levels and epilepsy in the propensity score-matched cohort.

Serum uric acid, μmol/L	Model 1		Model 2		Model 3	
	OR (95%CI)	*p*-value	OR (95%CI)	*p*-value	OR (95%CI)	*p*-value
Q1	1.79 (1.48, 2.16)	<0.0001	1.98 (1.64, 2.40)	<0.0001	1.94 (1.56, 2.42)	<0.0001
Q2-3	Ref		Ref		Ref	
Q4	1.39 (1.14, 1.69)	<0.0001	1.02 (0.83, 1.26)	0.82	1.33 (1.02, 1.74)	0.03

Model 1: No covariates were adjusted.

Model 2: Age and gender were adjusted.

Model 3: Age, gender, NIHSS, Uremia, Deep vein thrombosis, Fatty liver, Diabetes, Hypertension, Coronary disease.

Atrial fibrillation, Cerebral herniation, Hydrocephalus, Hyperlipidemia, Hypoproteinemia, Anterior cerebral artery.

Middle cerebral artery, Posterior cerebral artery, Vertebral artery, Basilar artery, Platelet count, Red blood cell count.

LDL-C, Creatinine, Urea, and Fibrinogen were adjusted.

Q, quartiles; OR, odds ratio; 95% CI, 95% confidence interval.

### Sensitivity analysis

To ensure the robustness of our findings and exclude potential confounding effects, a sensitivity analysis was conducted by excluding patients with uremia, hydrocephalus, or cerebral herniation - conditions that might influence SUA levels and/or epilepsy risk (22–24). The results, presented in Supplementary Table S5, remained consistent with the primary analysis. Univariate analysis showed a significant association between SUA quintiles and PSE, with SUA Q1 and Q4 exhibiting elevated ORs compared to the reference group (Q3). Specifically, SUA Q1 had an OR of 2.12 (95% CI: 1.97–2.59, *p* < 0.0001), while SUA Q4 had an ORs of 1.59 (95% CI: 1.29–1.97, p < 0.0001). Multivariable analysis revealed adjusted ORs of 2.52 (95% CI: 1.95–3.24, p < 0.0001) for SUA Q1 and 1.68 (95% CI: 1.33–2.12, p < 0.0001) for SUA Q4, further corroborating the U-shaped relationship observed in the main analysis.

## Discussion

The present study provides compelling evidence of a U-shaped relationship between SUA levels and the risk of PSE in patients with acute ischemic stroke. Using data from a large cohort, we demonstrated that both low (Q1: <305.3 μmol/L) and high (Q4: >373.9 μmol/L) SUA levels were associated with significantly increased risks of PSE compared to the moderate levels (Q2-Q3: 305.3–373.9 μmol/L). After adjusting for multiple confounders, including demographic factors, stroke severity (NIHSS score), comorbidities, and laboratory markers, the adjusted ORs for the Q1 and Q4 groups were 2.32 (95% CI: 1.79–2.99) and 1.60 (95% CI: 1.26–2.02), respectively. This pattern was confirmed through RCS analyses, which identified an inflection point at 335.3 μmol/L. Moreover, the robustness of these findings was reinforced by PSM and sensitivity analyses. After PSM, the non-linear association remained statistically significant. Notably, a significant interaction between SUA quartiles and sex was observed (P for interaction < 2.2 × 10^−16^), suggesting that the overall association reflects an average effect and is more pronounced in men (Supplementary Tables S1, S2). Given the close link between SUA and renal function, we adjusted for urea, creatinine, and uremia (Model 3) and performed PSM; however, slight post-matching imbalance in renal biomarkers remained (Supplementary Figure S1), and residual confounding cannot be excluded. Collectively, our results underscore the dual role of SUA as both a potential protective factor and a risk factor for PSE, depending on its concentration.

This non-linear relationship can be mechanistically explained by the dual biological properties of uric acid (16, 25). On one hand, SUA acts as a potent endogenous antioxidant, capable of scavenging reactive oxygen species (ROS) and inhibiting lipid peroxidation (26, 27). This antioxidant capacity is particularly relevant in the context of ischemic stroke, where oxidative stress contributes significantly to neuronal damage and subsequent epileptogenesis (28). Moderate SUA levels may thus confer neuroprotection by mitigating oxidative injury, thereby reducing the likelihood of PSE. On the other hand, at high concentrations, SUA can crystallize and trigger inflammatory pathways, such as the NLRP3 inflammasome, leading to the release of pro-inflammatory cytokines (e.g., IL-1β, IL-18) (29). Chronic inflammation is a known driver of epileptogenesis, as it promotes neuronal hyperexcitability and synaptic reorganization (30–32). Additionally, elevated SUA is often linked to metabolic disorders (e.g., hyperuricemia, gout) and renal impairment (15, 33), which may independently exacerbate stroke outcomes and increase susceptibility to seizures.

Conversely, very low SUA levels may reflect a state of compromised antioxidant defense (34). Inadequate ROS scavenging could leave neurons vulnerable to oxidative damage, facilitating excitotoxic neuronal death and aberrant synaptic plasticity—key processes in epilepsy development (35, 36). The identified inflection point (335.3 μmol/L) is consistent with this dual-role hypothesis, suggesting that the balance between antioxidant and pro-inflammatory milieus may vary across SUA levels.

Clinically, these findings suggest that SUA could serve as a valuable biomarker for PSE risk stratification. Patients with SUA levels outside the range associated with the lowest predicted odds in our data may warrant further investigation in future studies. For instance, low and high SUA levels may reflect different underlying biological profiles (e.g., oxidative stress–related vs. inflammatory/metabolic states). These mechanistic hypotheses require validation in experimental studies and prospective cohorts.

Our results both corroborate and extend the existing literature on SUA and neurological outcomes (37). Several studies have highlighted the neuroprotective role of SUA in stroke recovery. For example, Chamorro et al. (38) reported that higher SUA levels were associated with improved functional outcomes after acute ischemic stroke, attributing this to SUA’s antioxidant properties. Similarly, Dhanesha et al. (39) found that SUA administration reduced infarct size and improved neurological recovery in animal models of stroke. These studies support our observation that moderate SUA levels (Q2-Q3) are protective against PSE.

However, our discovery of increased PSE risk at high SUA levels appears to contradict some prior reports. This discrepancy may stem from differences in outcome measures: while earlier studies focused on short-term functional recovery, our work specifically examined PSE-a long-term complication influenced by chronic inflammation and neuronal hyperexcitability. Importantly, recent evidence has begun to acknowledge the “paradoxical” role of SUA. Zhang et al. (40) reported that high SUA levels were associated with increased risk of post-stroke complications, including epilepsy. Similarly, Weir et al. (41) identified SUA as a predictor of poor outcomes in stroke patients, particularly among those with pre-existing comorbidities. Wang et al. demonstrated that aberrant serum uric acid levels, whether excessively elevated or markedly reduced, are associated with an increased risk of epilepsy secondary to cerebral infarction, supporting SUA as a clinically relevant biomarker associated with PSE risk (14). These studies highlight the complex interplay between SUA, inflammation, and oxidative stress in the post-stroke brain.

Unique to our study is the characterization of this non-linear association using flexible modeling (RCS) and robustness checks (PSM and sensitivity analyses), which strengthens confidence that the pattern is not solely driven by measured confounding. In contrast to prior research that primarily focused on overall stroke outcomes, our study specifically investigates PSE, a clinically significant but underexplored complication of stroke.

While our study provides important insights, several limitations must be acknowledged. First, the observational design limits our ability to infer causality between SUA levels and PSE risk. Second, despite PSM and multivariable adjustments, slight post-matching imbalance in renal biomarkers persisted (Supplementary Figure S1), and confounding by renal function and other unmeasured factors cannot be fully ruled out. Third, excluding patients who died in the ultra-early phase may shift the sample toward survivors with milder disease, thereby limiting the generalizability of the observed SUA–PSE association. Likewise, excluding patients with severe comorbidities (e.g., uremia, hydrocephalus, or cerebral herniation) may further limit the applicability of our findings to broader stroke populations, as these patients may exhibit distinct SUA–PSE dynamics. Additionally, SUA levels were measured at a single time point, which does not capture potential temporal fluctuations or the cumulative effects of SUA over time. Longitudinal studies are needed to better understand how changes in SUA levels influence PSE development. Moreover, the study population was derived from a single center, which may limit the external validity of our findings to other regions or healthcare settings. Finally, the original publication did not report detailed diagnostic criteria for post-stroke epilepsy (e.g., EEG confirmation, ICD coding rules, exclusion of acute symptomatic seizures) or a prespecified adjudication workflow; therefore, we used the dataset-labeled outcome as provided. This may introduce outcome misclassification, which should be considered when interpreting the estimated associations. Future research should aim to elucidate the mechanisms underlying the U-shaped relationship between SUA and PSE through experimental and longitudinal studies. Investigating the role of SUA modulation in animal models of stroke and epilepsy could provide valuable mechanistic insights. Additionally, randomized clinical trials evaluating interventions to optimize SUA levels in stroke patients are warranted. Expanding the study population to include diverse stroke subtypes and severity levels will enhance the generalizability of future findings. Finally, integrating genetic, metabolic, and dietary data could provide a more comprehensive understanding of the factors influencing SUA-PSE dynamics.

## Conclusion

Our study builds on existing evidence and provides novel insights into the association between serum uric acid (SUA) and post-stroke epilepsy (PSE). We observed a non-linear relationship, with the lowest predicted odds of PSE at intermediate SUA levels, suggesting that SUA may reflect heterogeneous biological states relevant to post-stroke neuroinflammation and oxidative stress. These findings are hypothesis-generating and warrant validation in prospective cohorts and mechanistic studies.

## Data Availability

Publicly available datasets were analyzed in this study. This data can be found at: https://doi.org/10.5061/dryad.w0vt4b92c.

## Footnotes

1
https://doi.org/10.5061/dryad.w0vt4b92c
