# Supplementary material for: U-shaped association between serum uric acid at admission and post-stroke epilepsy in patients with ischemic stroke: a cohort study
Source: Front Neurol. 2026 Feb 19;17:1759537. doi: 10.3389/fneur.2026.1759537 (PMC12960181; doi:10.3389/fneur.2026.1759537)

Supplementary Materials

Table S1 Variance inflation factor (VIF)

Variable	GVIF	DF	GVIF^(1/(2*DF))
buaQ	2.084183064	3	1.130201754
Gender	2.009499625	1	1.417568208
Age	1.858596395	1	1.363303486
Uremia	1.511282866	1	1.229342453
Deep vein thrombosis	1.159367852	1	1.076739454
Fatty liver	1.158509213	1	1.076340659
Diabetes	1.246149194	1	1.116310528
Hypertension	1.378611314	1	1.1741428
Coronary_disease	1.316454223	1	1.14736839
Atrial_fibrillation	1.217522728	1	1.103414123
Cerebral_hernia	1.082401719	1	1.04038537
Hydrocephalus	1.106454225	1	1.051881279
Hyperlipidaemia	1.150513869	1	1.072620095
Hypoproteinemia	1.581937488	1	1.257750964
Platelet count	1.574392566	1	1.254748009
Red blood cell count	2.497757646	1	1.580429576
LDL-C	1.191274786	1	1.091455352
Fibrinogen	1.866366309	1	1.366150178
Urea	1.658031502	1	1.287645721
Creatinine	1.853676464	1	1.361497875
Anterior cerebral artery	1.045891588	1	1.022688412
Middle cerebral artery	1.107767013	1	1.052505113
Posterior cerebral artery	1.030420875	1	1.015096486
Vertebral artery	1.541566454	1	1.241598346
Basilar artery	1.501559352	1	1.225381309
NIHSS	1.528838975	1	1.236462282

Table S2 Association between serum uric acid (SUA) quartiles and PSE: sex-stratified multivariable logistic regression

character	Q3	Q1 (OR, 95% CI)	p	Q2 (OR, 95% CI)	p	Q4 (OR, 95% CI)	p	p for interaction
Gender								< 0.0001
Female	ref	1.074(0.794,1.472)	0.651	0.838(0.602,1.176)	0.299	1.341(0.934,1.931)	0.112	
Male	ref	5.740(4.436,7.463)	<0.0001	2.315(1.772,3.035)	<0.0001	1.747(1.364,2.250)	<0.0001	

Table S3 Likelihood ratio test for interaction between SUA quartiles (buaQ) and gender on PSE

Model	Residual df	Residual deviance	Δdf	LR χ^2 (ΔDeviance)	P for interaction
Model 1 (main effects)	21430	5832.1	—	—	—
Model 2 (with interaction)	21427	5727.3	3	104.83	$<2.2\times10^{-16}$

Model 1: Adjusted for Age, gender, NIHSS, Uremia, Deep vein thrombosis, Fatty liver, Diabetes, Hypertension, Coronary disease, Atrial fibrillation, Cerebral herniation, Hydrocephalus, Hyperlipidemia, Hypoproteinemia, Anterior cerebral artery, Middle cerebral artery, Posterior cerebral artery, Vertebral artery, Basilar artery, Platelet count, Red blood cell count, LDL-C, Creatinine, Urea, and Fibrinogen.

Model 2: Included the interaction term between SUA quartiles (buaQ) and gender (buaQ×gender), and adjusted for the same covariates as Model 1.

Table S4 Standardized Mean Differences (SMD) of Covariates before and after Propensity Score Matching

	Q1 vs Q2-3		Q4 vs Q2-3		
	Unweight	Weighted	Unweight	Weight	Threshold
Anterior cerebral artery	0.135377599	0.094897067	0.007426379	0.003734959	<0.2
Age	0.186113694	0.170602078	0.06085303	0.005920317	<0.2
Atrial fibrillation	0.000400772	0.00465959	0.117186284	0.054272223	<0.2
Basilar artery	0.09980938	0.054741751	0.032950337	0.013580078	<0.2
Cerebral hernia	0.123868832	0.080320187	0.014269457	0.015522595	<0.2
Coronary disease	0.122716297	0.015032604	0.275868864	0.098992328	<0.2
Creatinine	0.342602258	0.043979078	0.375862699	0.309660538	<0.2
Diabetes	0.316964151	0.099657603	0.202771663	0.147132615	<0.2
Deep vein thrombosis	0.149952784	0.076476490	0.048507775	0.006172097	<0.2
Fatty liver	0.225180498	0.147596594	0.234948855	0.170719013	<0.2
Fibrinogen	0.001761847	0.141994891	0.305023662	0.183676425	<0.2
Gender	0.627175908	0.213087668	0.444422259	0.018505956	<0.2
Hydrocephalus	0.006973559	0.019210654	0.032160906	0.016507566	<0.2
Hyperlipidaemia	0.04003139	0.064090632	0.137330174	0.089987720	<0.2
Hypertension	0.106660825	0.049512849	0.417501328	0.138867679	<0.2
Hypoproteinemia	0.380960685	0.249029717	0.017772048	0.045590050	<0.2
Low-density lipoprotein	0.13552663	0.106669562	0.026885465	0.064832405	<0.2
Middle cerebral artery	0.012375998	0.008626229	0.001539682	0.031792630	<0.2
NIHSS	0.335530023	0.207508202	0.289742137	0.103200307	<0.2
Posterior cerebral artery	0.000110236	0.009933300	0.024758805	0.016484092	<0.2
Platelet count	0.056993993	0.122399698	0.116562763	0.007572381	<0.2
Red blood cell count	0.475867896	0.140376188	0.238464646	0.027024287	<0.2
Urea	0.19313461	0.019287319	0.366535264	0.294283218	<0.2
Uremia	0.029924861	0.002980229	0.137067595	0.115808261	<0.2
Vertebral artery	0.041349471	0.031971453	0.030800578	0.014651150	<0.2

Table S5 Association Between Serum Uric Acid Levels and Epilepsy in Patients Excluding Uremia, Hydrocephalus, or Cerebral Herniation

	Model 1		Model 2		Model 3	
Serum uric acid, μmol/L	OR (95%CI)	*P*-value	OR (95%CI)	*P*-value	OR (95%CI)	*P*-value
Q1	2.12(1.73,2.59)	<0.0001	3.02(2.44,3.74)	<0.0001	2.52(1.95,3.24)	<0.0001
Q2	1.4(1.13,1.74)	0.002	1.73(1.39,2.16)	<0.0001	1.42(1.11,1.81)	0.01
Q3	Ref		Ref		Ref	
Q4	1.59(1.29,1.97)	<0.0001	1.53(1.24,1.89)	<0.0001	1.68(1.33,2.12)	<0.0001

Model 1: No covariates were adjusted.

Model 2: Age and gender were adjusted.

Model 3: Age, gender, NIHSS, Uremia, Deep vein thrombosis, Fatty liver, Diabetes, Hypertension, Coronary disease, Atrial fibrillation, Cerebral herniation, Hydrocephalus, Hyperlipidaemia, Hypoproteinemia, Anterior cerebral artery, Middle cerebral artery, Posterior cerebral artery, Vertebral artery, Basilar artery, Platelet count, Red blood cell count, Low-density lipoprotein, Creatinine, Urea, and Fibrinogen were adjusted.

Abbreviations: Q, quintile; OR, odds ratio; 95% CI, 95% confidence interval.

FIGURES AND FIGURE LEGENDS

Fig. S1 The absolute value of standardized mean difference of covariates before and after PSM. Abbreviations: SMD, standardized mean difference. (A) Unadjusted model. (B) Model adjusted for age and sex. (C) Fully adjusted model including age, sex, NIHSS at admission; comorbidities (uremia, deep vein thrombosis, fatty liver, diabetes, hypertension, coronary disease, atrial fibrillation, cerebral herniation, hydrocephalus, Hyperlipidaemia, Hypoproteinemia); major cerebral artery stenosis/occlusion (anterior, middle, posterior cerebral; vertebral; basilar); and laboratory measures (platelet count, red blood cell count, LDL-C, creatinine, urea, fibrinogen). Nonlinearity was assessed with a likelihood ratio test; *P*<0.001 for all panels.

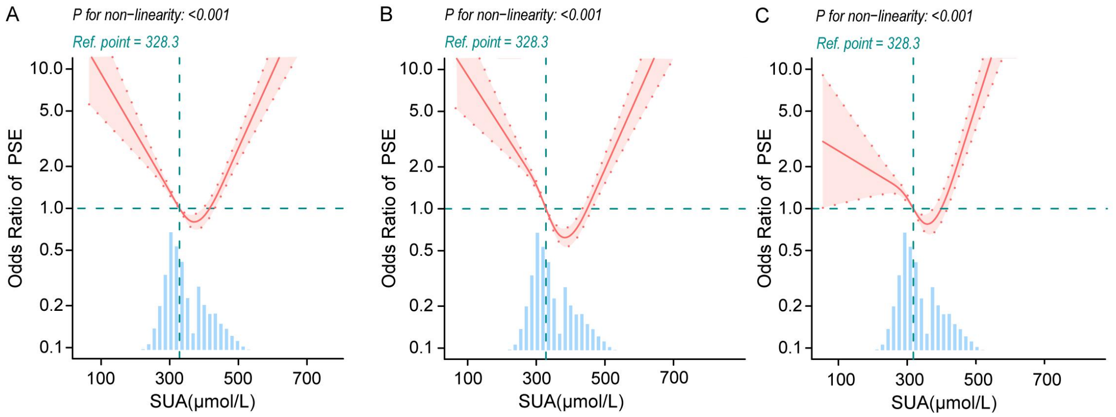

Fig. S2 Nonlinear association between serum uric acid at admission and post-stroke epilepsy (PSE) in the propensity score – matched cohort. The solid red curve denotes the estimated odds ratio (OR) and the shaded area the 95% confidence interval; the horizontal dashed line indicates OR=1, and the vertical dashed line marks the reference SUA level (328.3 μ mol/L). The histogram below shows the distribution of SUA. (A) Unadjusted model. (B) Model adjusted for age and sex. (C) Fully adjusted model including age, sex, NIHSS at admission; comorbidities (uremia, deep vein thrombosis, fatty liver, diabetes, hypertension, coronary disease, atrial fibrillation, cerebral herniation, hydrocephalus,

Hyperlipidaemia, Hypoproteinemia); major cerebral artery stenosis/occlusion (anterior, middle, posterior cerebral; vertebral; basilar); and laboratory measures (platelet count, red blood cell count, LDL-C, creatinine, urea, fibrinogen). Nonlinearity was assessed with a likelihood ratio test; *P*<0.001 for all panels.

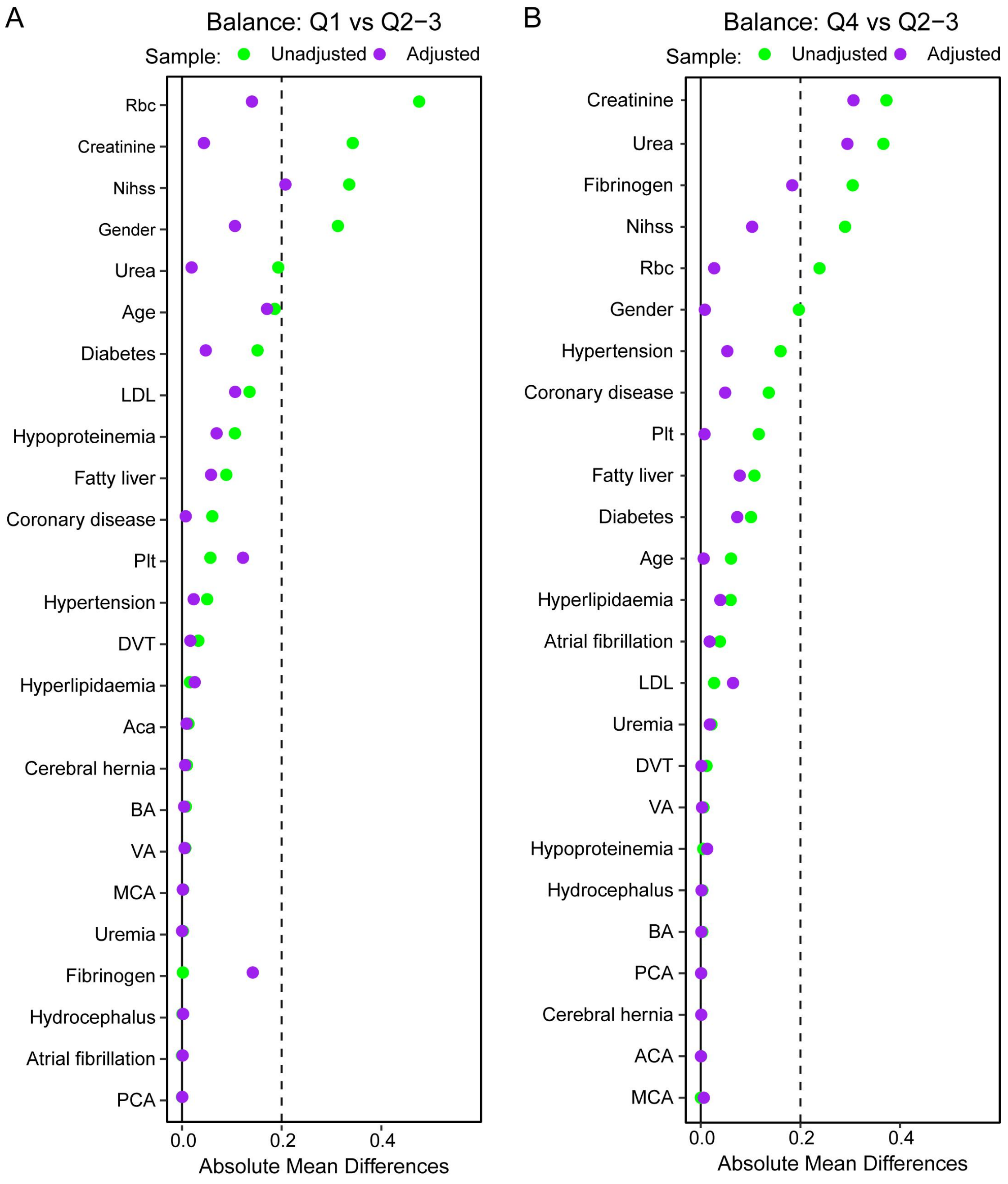

Supplement: Supplementary file 1 [file Image_1.pdf]
